# The pathogenicity comparison of *Lagovirus europaeus* GI.1 and GI.2 strains in China by using relative quantitative assay

**DOI:** 10.1038/s41598-022-25118-0

**Published:** 2022-11-28

**Authors:** Teng Tu, You Zhou, Dike Jiang, Maonan Pang, Xulong Wu, Xueping Yao, Yan Luo, Zexiao Yang, Meishen Ren, Yin Wang

**Affiliations:** 1 Key Laboratory of Animal Diseases and Human Health of Sichuan Province, College of Veterinary Medicine, Sichuan Agricultural University, Chengdu, 611130 China; 2 Branch of Animal Husbandry and Veterinary Medicine, Chengdu Agricultural College, Chengdu, 611130 China

**Keywords:** Virology, Animal physiology

## Abstract

*Lagovirus europaeus* GI.1 belongs to Lagovirus in the Caliciviridae family. GI.1 causes an acute, septic, and highly lethal disease in rabbits. *Lagovirus europaeus* GI.2, a new variant of GI.1, has caused explosive mortality in rabbits of all ages in Sichuan Province, China. To explore the differences in pathogenicity of rabbits infected with GI.1/GI.2, we investigated the virulence and disease progression of a naturally occurring GI.1/GI.2 in 4-week-old, 13-week-old, and 25-week-old New Zealand White laboratory rabbits after GI.1/GI.2 infection. Objective measures of disease progression were recorded using continuous body-temperature monitoring. We observed the kittens were infected with GI.2 during the most urgent course of the disease, and GI.1 was not lethal to kittens. We found that the target organ of both GI.1 and GI.2 was the liver, but the disease course of the two viruses was differed. Our study enriches the research on the pathogenicity of GI.1 and GI.2 under the same conditions.

## Introduction

Rabbit hemorrhagic disease (RHD) is also known as rabbit fever or hemorrhagic pneumonia. It is caused by two lagoviruses of the Caliciviridae family^1^. Consequent upon the 2017 attempt to standardise the Lagovirus nomenclature, a single species of lagovirus would be recognized and called *Lagovirus europaeus*, RHDV belongs to the GI genogroup, which is further divided into the GI.1(former G1–G6), GI.2 (RHDV2/b), GI.3 (nonpathogenic rabbit calicivirus E-1) and GI.4 (nonpathogenic rabbit calicivirus A-1/E-2) genogroups^2^. GI.1 was first reported in China in 1984^3^. South Korea was the following country to report RHD outbreaks due to the rabbit fur imported from China^4^. The disease then emerged in Europe and first reported in Italy in 1986^5^, from where it spread to other parts of Europe and became endemic in several countries. In Australia and New Zealand, rabbits are considered an important agricultural pest and a threat to animals and plants ^6^, and GI.1 has been used to control rabbit populations ^7^.

In 2010, the novel lagovirus GI.2, which is genetically and antigenically distinct from GI.1, was reported in wild and domestic rabbits in France and Spain ^8,9^. GI.1 and GI.2 are different genotypes of the lagovirus causing RHD. GI.2 can infect both rabbits and hares, and rabbits are more susceptible, especially kitten and subadult rabbits. Additionally, GI.2 causes disease in several hares (Lepus) species and can overcome GI.1 vaccine protection ^10–12^.

On April 1, 2020, an acute death case of rabbits occurred on a rabbit farm in Jintang, Sichuan Province, China. The clinical symptoms and pathological changes in the dead rabbits were very similar to those of RHD, and deaths occurred in rabbits of all ages. Rabbits were injected with GI.1, and Pasteurellosis multocida combined inactivated vaccine before and five days after the onset of rabbit farm, but the effect was not obvious^13^. In a previous study, we isolated a GI.2 strain SCCN04 from these dead rabbits at a rabbit farm in Jintang ^14^.

According to the results published by Abrantes et al. ^15^, their results indicate that recombination contributed to the emergence, persistence and dissemination of GI.2 as a pathogenic form and that all described GI.2 strains so far are the product of recombination. In addition, with pathogenic (variants GI.1a and GI.1b) and benign (genotype GI.4) strains that served as donors for the non-structural part while GI.2 composed the structural part. In our previous study^14^, we performed genetic characterization of SCCN04, where the whole genome phylogenetic tree, the non-structural phylogenetic genes tree and the structural genes phylogenetic tree were constructed, and recombination events were analysed. The results showed that SCCN04 and Chinese isolate SC2020/0401(MT586027.1) formed a small branch and were the most closely related among all the genome phylogenetic trees, and SCCN04 was closely related to European GI.2 isolates on a large branch. In addition, we confirmed SCCN04 recombination by RDP5 and identified 2020 isolate MT586027.1 (SC2020/0401) as the main parental virus. This indicates that GI.2 may have a new restructuring event in China.

There had been many previous studies dealing with the pathogenicity of GI.1 or GI.2^16–19^, and pointing out some differences in the pathogenicity of the two viruses, but these studies were not all conducted under the same conditions, so the results are not directly comparable. Our study reports a systematic comparison, control the relevant parameters of GI.1/GI.2 challenge experiment uniformly (both viruses simultaneously, rabbit age groups, same virus dose and inoculation route). To compare the difference in pathogenicity between GI.2 and GI.1, GI.2 (SCCN04) and GI.1 (SCH04) were used to infect rabbits of various ages, respectively. Changes in the virus copies in blood, feces, and saliva over time after infection with GI.1/GI.2 were detected by RT-qPCR established according to the VP60 gene, because the VP60 gene of the same genotype isolates (GI.1or GI.2) was highly conserved. The β-actin gene was used as a reference gene, and the GI.1 and GI.2 RT-qPCR methods established in our laboratory^20,21^ were used to establish a relative quantitative assay to detect the GI.1 and GI.2 load distribution in rabbits on different days. SCCN04 (Accession: MW178244.1) and SCH04 (Accession: KX844830.1) were isolated from dead rabbits in Chengdu, Sichuan Province, China, preserved, and provided by Sichuan Agricultural University.

## Materials and methods

### Animal trials

GI.1 and GI.2-infected animals trials included three identical groups (S, M, and L groups), respectively. Group S included 4-week-old kitten (range 28–30 days at infection, weight 512–734 g [${\overline{\text{X}}} = 631$ g], 4 males, 5 females). Group M included 13-week-old subadult rabbits (range 89–91 days at infection, 1890–2197 g [${\overline{\text{X}}} = 2016$ g], 4 males, 5 females), and Group L included 25-week-old adult rabbits (range 172–184 days at infection, 6490–7831 g [${\overline{\text{X}}} = 6571$ g], 4 males, 5 females) New Zealand White rabbits were obtained from the non-immunized rabbit farm of rabbit breeders in Yaan, Sichuan Province, and all experimental rabbits were kept in a closed enclosure. The infection groups contained 6 animals (males and females half) per age. The infection doses of GI.1 and GI.2 were 1000 RID_50_, and 3 rabbits of each group were selected for the control group.

All animal experiments were approved by the Institutional Animal Care and Use Committee of Sichuan Agricultural University of China (Approval number: SYXK2019-187). All animal experiments were performed in accordance with the Laboratory Animals Welfare and Ethics guidelines published by the General Administration of Quality Supervision, Inspection, and Quarantine of the People’s Republic of China.

### Virus inoculum

The virus stocks were prepared by the Key Laboratory of Animal Diseases and Human Health of Sichuan Province. Briefly, the virus was amplified in New Zealand White rabbits, liver homogenates were semi-purified, and freeze-dried virus stocks were prepared and titrated to derive the 50% infectious rabbit dose (RID_50_) of the virus stock. For titration, groups of six adult rabbits were inoculated intramuscularly with10-fold serial dilutions of the concentrated virus stock, and the Reed-Muench method was used to determine the 50% endpoint^22^. The RID_50_ measurement results are shown in Table 1, the RID_50_ of GI.2 and GI.1 are 10^6.5^ and 10^5.5^ respectively. Rabbits were infected orally with a dose (1000 RID_50_) of the virus, in a final volume of 1 mL.

**Table 1:** RID_50_ results of GI.2 and GI.1 strains.

Genotype	Strains	RID_50_
GI.2	SCCN04	10^6.5^
GI.1	SCH04	10^5.5^

*RID* rabbit infective dose.

### Monitoring

Monitoring of temperature and activity began the day before infection to obtain baseline recordings for each individual. Body temperature was measured as rectal temperature. To ensure the data were accurate, 6 researchers measured each rabbit's rectal temperature every three hours after infection, day and night. In this trial, humane endpoints were established to minimize disease and suffering while still observing as complete a disease course as possible to evaluate the welfare impacts of infection. Rabbits with a terminal disease, most obviously assessed by a rectal temperature less than 38 °C with lethargy and anorexia, were humanely killed by intravenous barbiturate overdose after sedation with xylazine (5 mg/kg) and ketamine 30 mg/kg administered intramuscularly.

### Sample collection

Blood, saliva, and feces were collected before infection. Samples of blood, saliva, and feces were collected every 3 h during the first 12 h of infection, and every 6 h thereafter. Specifically, 2 mL of blood was collected through the ear vein, saliva was dipped into the rabbit's mouth with cotton swabs, and fecal particles were collected. Due to the rapid course of RHDV infection, animals died peracute between monitoring timepoints. Therefore, when we found more than 80% of the rabbits appeared to have terminal disease at monitoring timepoints in a group, all rabbits in this group were killed humanely. At the same time, the rabbits in the control group were sacrificed and dissected. Samples of the liver, spleen, lung, heart, and kidney were collected sequentially from each dead rabbit. RNA was extracted from each tissue sample and reverse-transcribed into cDNA, and GI.1/GI.2 RT-qPCR was performed. The viral RNA extraction kit and reverse transcription kit were purchased from TaKaRa (Beijing, China). Genious2 × SYBR Green Fast qPCR Mix (No ROX) was purchased from ABclonal (Wuhan, China).

### Analysis of relative gene expression data using RT-qPCR and the 2^−ΔΔCT^ method

β-Actin gene (GenBank accession number: NM_001101683.1) was stably expressed in all organs of rabbits, and the Ct value of the β-actin gene was detected using the RT-qPCR method established by Chen Rui^23^. The GI.1 and GI.2 RT-qPCR methods established in our laboratory were used to detect the CT values of the target genes. Ct values for all genes were recorded, and the relative viral load was calculated using the 2^−ΔΔCT^ method^24^. All primers used are listed in Table 2, and Details of the RT-qPCR methods are shown in Table S1. In order to verify that the extracted amplicon was the correct target, we conducted three repetitions of each sample for verification in the qPCR experiment, and only when all three repetitions were amplified, melting curves are identical and the target gene is amplified by agarose gel electrophoresis could we determined that the correct target was amplified. In addition, to ensure that the qPCR does not amplify false positives, Evaluation was repeated if a typical amplification curve was observed with a Ct value of greater than 38. Samples that underwent repeated tests with the same result were considered positive, and those showing inconsistent results in repeated tests were considered negative.

**Table 2:** The sequences of primer.

Primers	Primers sequence (5′–3′)	Target gene	Annealing temperature (°C)	Fragment length
GI.2-F	AACCACTGCCGGTGACAG	VP60	55.4	203
GI.2-R	ATCAACACTCAAGCCAAG			
GI.1-F	TGGAGATYGGTTTRAGTG	VP60	57.1	165
GI.1-R	AATGAGTTCAGTCARGTCAA			
*β-actin*-F	CAAGCGTGGCATCCTGAC	*β-actin*	60	100
*β-actin*-R	CTCGTTGTAGAAGGTGTGGTG			

All Statistical analysis was performed with GraphPad Prism 8.0.2 (GraphPad Software, San Diego, CA, USA), and all data were analyzed by one-way ANOVA.

### Preparation and scoring of pathological sections

In animal trials, all rabbits' (control and infected groups) hearts, livers, spleens, lungs, and kidneys were soaked in 4% paraformaldehyde and sent to Sevicebio Company for section preparation and pathological scoring. Pathological changes were scored on a five-point scale, with no or very few lesions scored as 0; mild or few lesions were scored as 1; moderate lesions or moderate lesions were scored as 2; severe or multiple lesions were scored as 3; extremely serious or massive lesions were scored as 4.

### Ethics approval

All animal experiments were approved by the institutional animal care and use committee of Sichuan Agricultural University of China (Approval number: SYXK2019-187). All animal experiments were performed under the Laboratory Animals Welfare and Ethics published by the General Administration of Quality Supervision, Inspection, and Quarantine of the People’s Republic of China. And the study is reported in accordance with ARRIVE guidelines (https://arriveguidelines.org).

## Results

### Clinical manifestations after infection

The kittens in the GI.1-S group showed no obvious clinical symptoms after inoculation, and they were sacrificed at 72 h post-infection (hpi). The other infected rabbits died within 3 days after inoculation. The death time of rabbits in the GI.2-S group was the shortest, with all kittens dying within 18 h. All the dead rabbits showed the typical clinical symptoms of RHD. Specific manifestations included: depression, loss of appetite, sudden death, violent struggle before death, angular arch retraction, and oral bleeding in some rabbits.

### Temperature changes preceding death following GI.1/GI.2 infection

**Figure 1:**
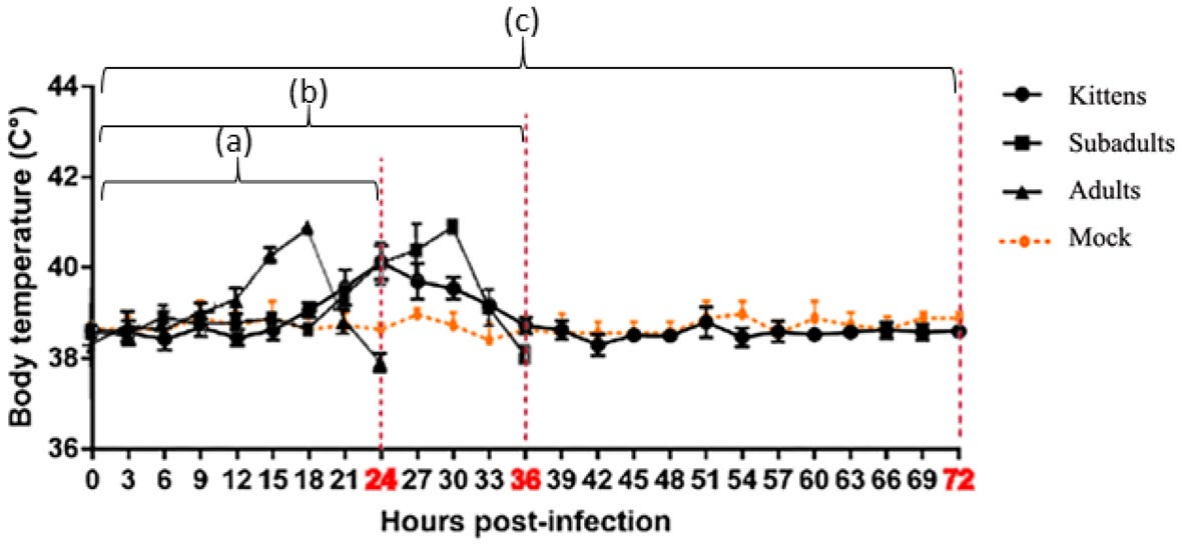
Temperature changes preceding death following GI.1 infection. To ensure the data were accurate, 6 researchers measured each rabbit's rectal temperature every three hours after infection, day and night. Mean temperature for timepoint is listed with the standard deviation. Red dotted lines indicate when rabbits were sacrificed/died at different timepoints. (a) Body temperature changes before the adults’ humane endpoint (at 24 hpi); (b) Body temperature changes before the subadults’ humane endpoint (at 36 hpi); (c) Body temperature changes before the kittens were sacrificed (at 72 hpi).

As shown in Fig. 1, after GI.1 infection in rabbits, pyrexia peaked 6 h, on average, prior to the endpoint (range 4–12 h before the endpoint) in both adult and subadult rabbits. The body temperature of kittens increased within 24 h and then tended to stabilize. The surviving kittens in the infection experiment were killed manually at 72 h to determine visceral viral load.

**Figure 2:**
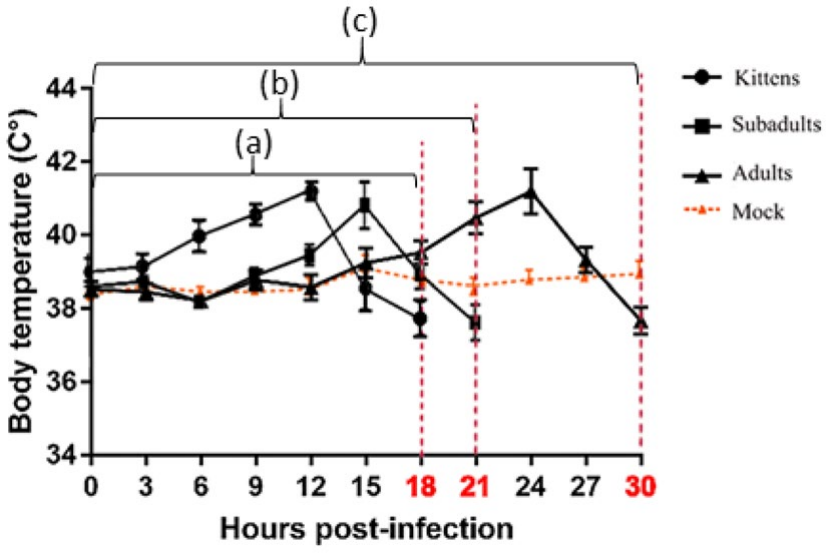
Temperature changes preceding death following GI.2 infection. To ensure the data were accurate, 6 researchers measured each rabbit's rectal temperature every three hours after infection, day and night. Mean temperature for timepoint is listed with the standard deviation. Red dotted lines indicate when rabbits were sacrificed/died at different timepoints. (a) Body temperature changes before the kittens’ humane endpoint (at 18 hpi); (b) Body temperature changes before the subadults’ humane endpoint (at 21 hpi); (c) Body temperature changes before the adults’ humane endpoint (at 30 hpi).

As shown in Fig. 2, after GI.2 infection in rabbits, pyrexia peaked on average 6 h before the endpoint (range 4–10 h before the endpoint) in all groups. Unlike GI.1 infection, GI.2 was lethal to kittens, who died the fastest and experienced the fastest rise in body temperature after infection with GI.2.

### Kinetics of viremia after GI.1 infection

**Figure 3:**
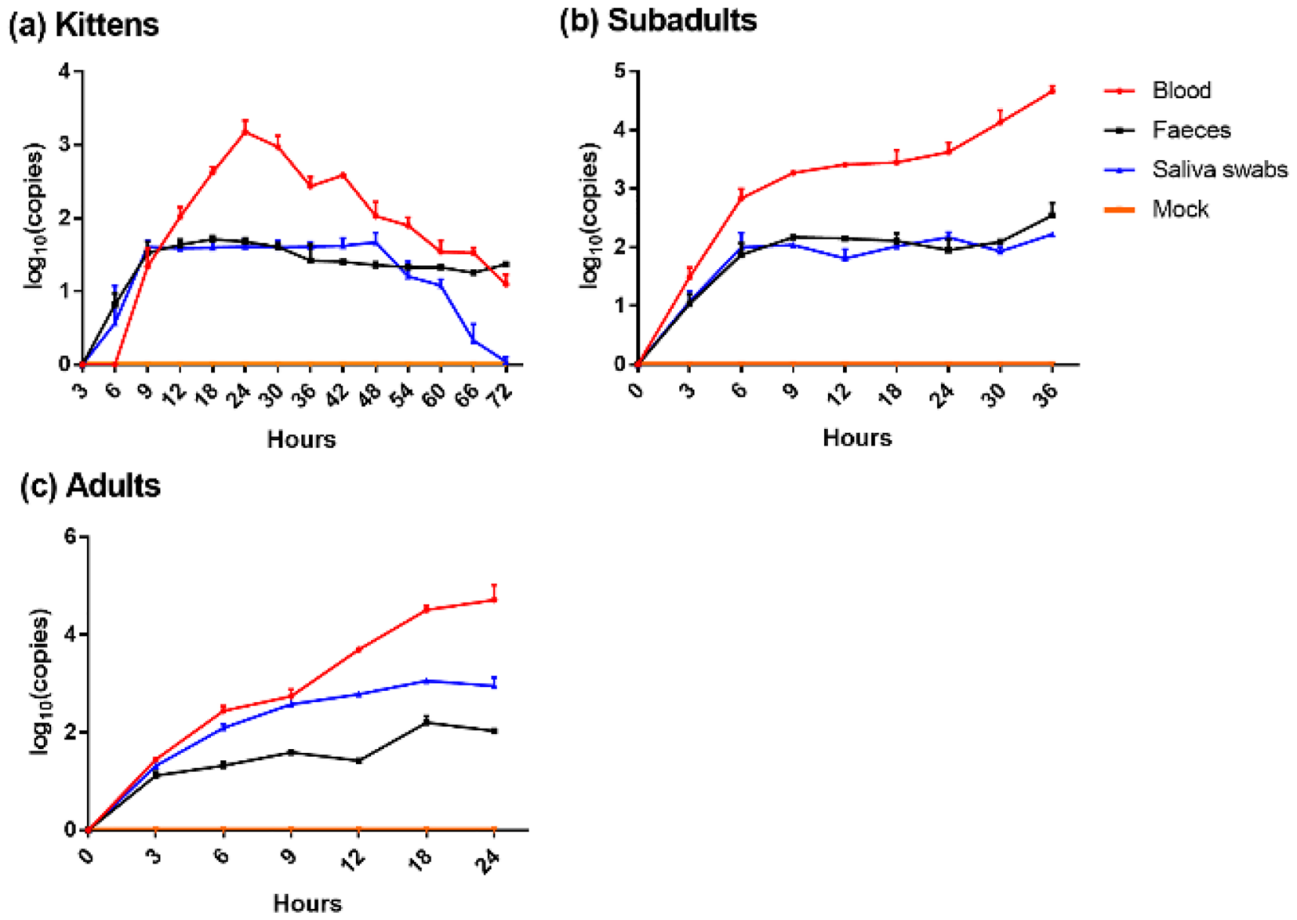
Viral copies in blood, feces, and saliva over time after GI.1 infection. The red, black, and blue broken lines represent the dynamic changes of viral copies in blood, feces, and saliva, respectively. Mean value is listed with the standard deviation. The orange line represent the control group, and the viral copies of samples tested at each timepoint was 0. After GI.1 infection, (**a**) for kittens, the viral copies in blood and saliva increased first and then decreased, while the viral copies in feces remained stable; while the viral copies in blood, saliva and feces in (**b**) subadults and (**c**) adults showed an increasing and then stabilizing trend.

The kinetics of viremia after GI.1 infection are shown in Fig. 3. The viral copies in the blood of kittens began to rise at 6 hpi, reached a maximum at 24 hpi, and then continued to decline. The viral copies in the blood of other rabbits increased rapidly within 6 hpi and then slowed.

The viral copies in the feces of kittens and subadult rabbits continued to increase within 9 hpi, and then tended to stabilize. The viral copies in the feces of adult rabbits increased within 3 hpi, then tended to stabilize, and then increased again after 12 hpi.

The viral copies in the saliva of kittens increased within 9 hpi, then plateaued, and began to decrease after 48 hpi. At 72 hpi, GI.1 was not detected. The viral copies in the saliva of subadult rabbits and adult rabbits first increased and then tended to be stabilize.

### Kinetics of viremia after GI.2 infection

**Figure 4:**
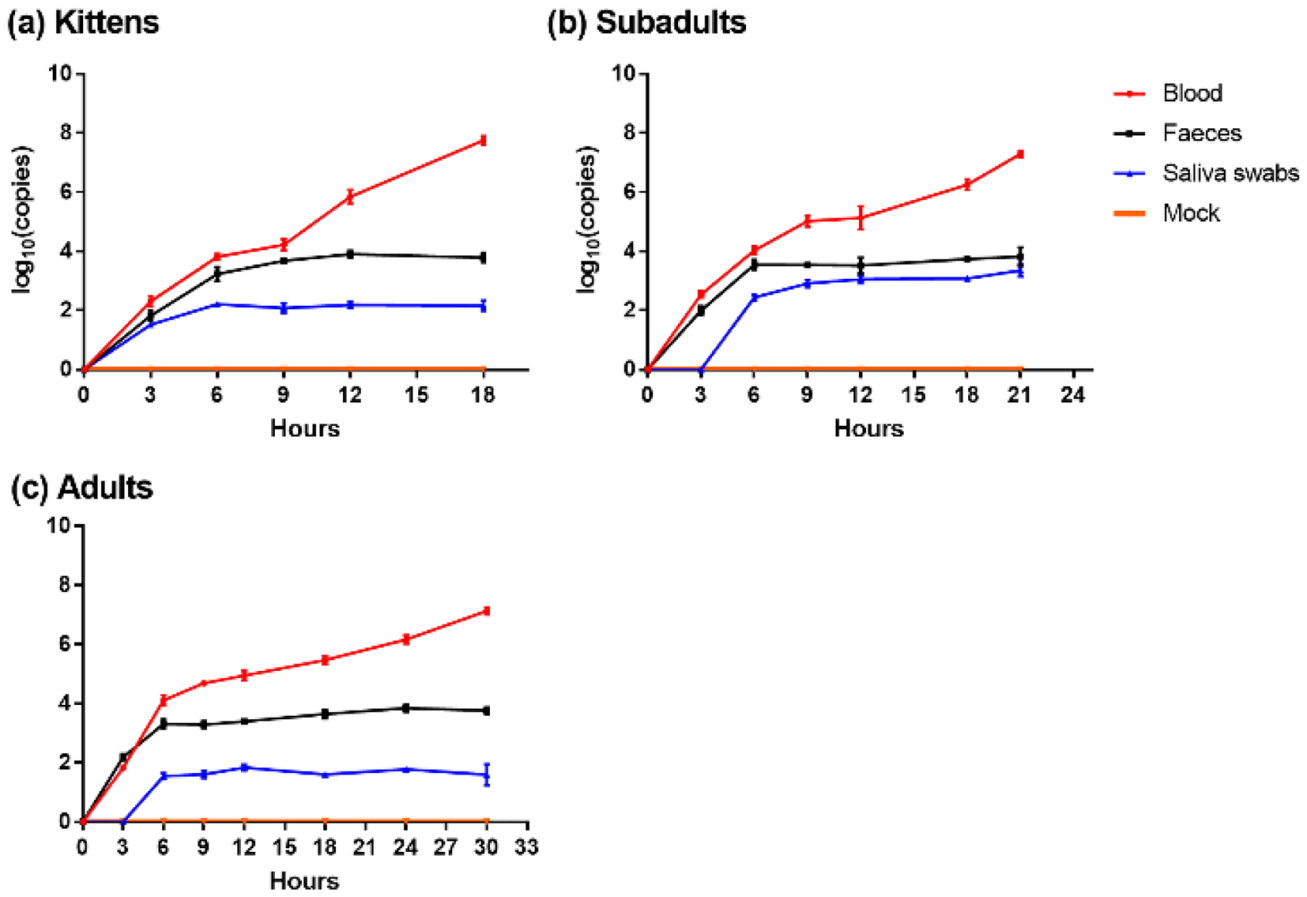
Viral copies in blood, feces, and saliva over time after GI.2 infection. The red, black, and blue broken lines represent the dynamic changes of viral copies in blood, feces, and saliva, respectively. Mean value is listed with the standard deviation. The orange line represent the control group, and the viral copies of samples tested at each timepoint was 0. After GI.2 infection, all rabbits [(**a**) kittens, (**b**) subadults, (**c**) adults] had an increased viral copies in blood, while the viral copies in feces and saliva remained stable after 6 hpi.

The kinetics of viremia after GI.2 infection are shown in Fig. 4. The viral copies in the blood of all rabbits increased rapidly, but the trend was slower in adult rabbits, and the time of adult rabbit death was later. The viral copies in the feces of all rabbits increased within 6 hpi, and then stabilized.

After 6 hpi, the viral copies in the saliva of all rabbits increased to a stable level. The virus was detected in the saliva of kittens within 3 hpi, whereas it was detected in the saliva of subadult and adult rabbits after 3 hpi.

### Visceral viral load and pathological score

The HE (Hematoxylin and Eosin) staining results of the rabbit viscera are shown in Supplementary Figures S1–S7. By combining with the score table of pathological sections of rabbits in the challenge group (Supplementary Tables S2–S6) and the distribution of visceral viral load of rabbits in the challenge group (Supplementary Tables S7–S8), a bar chart of visceral viral load and a pathological score of rabbits in the challenge group were drawn. Meanwhile, HE staining and GI.1/GI.2 detection were also performed on the visceral tissues of rabbits in the control group, and the results showed that no pathological changes were detected in the visceral tissues and no GI.1 or GI.2 was detected.

### GI.1 challenge

**Figure 5:**
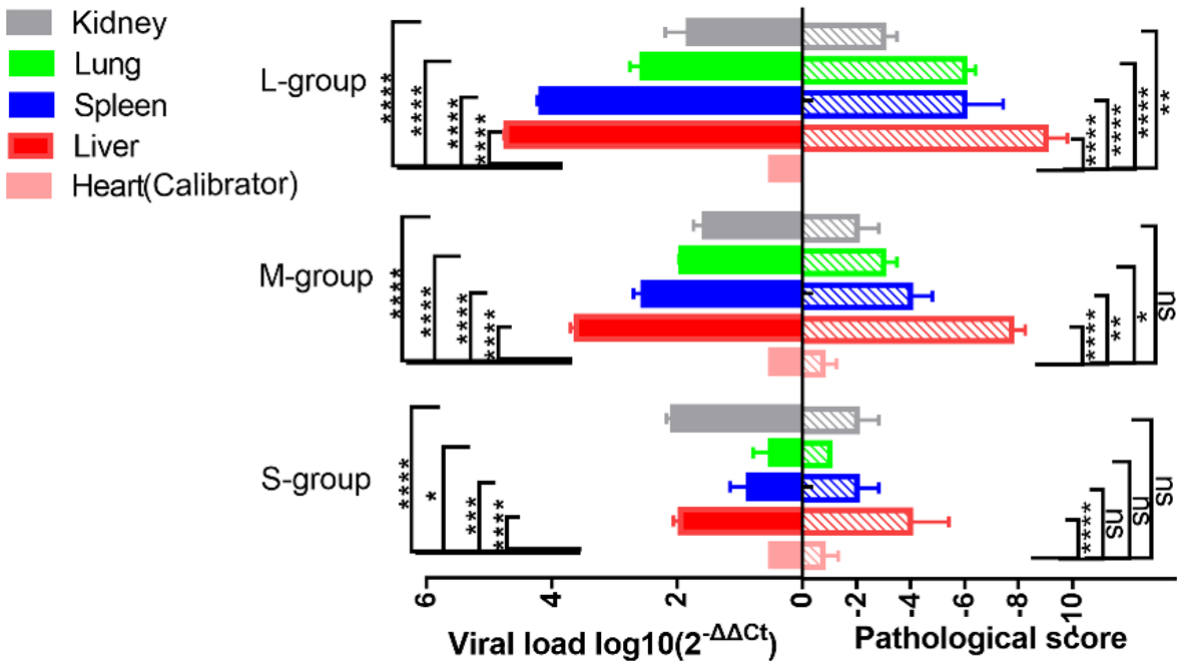
Column representation of visceral viral loads and a pathological scores of rabbits infected with GI.1. The viral load was calculated according to the formula 2^−ΔΔCT^. The higher the ΔCt value is, the lower the 2^−ΔΔCT^ value is. The heart with the highest ΔCt value and the lowest expression of GI.1 was selected as Calibrator. Different asterisks indicate significant differences, *(*p* < 0.05), **(*p* < 0.01), ***(*p* < 0.001), ****(*p* < 0.0001); ns means the difference is not significant (*p* > 0.05).

As shown in Fig. 5, there was a positive correlation between the viral loads and lesion scores of the viscera after GI.1 challenge. No apparent lesions were found in the hearts of rabbits in any of the experimental groups, and the viral load was the lowest. The liver, spleen, and lung tissue viral loads of adult rabbits were higher than those of kittens and subadult rabbits, and the lesion score was higher than that of kittens and subadult rabbits. There were no significant differences in the renal viral load or renal lesion scores among the rabbits.

### GI.2 challenge

**Figure 6:**
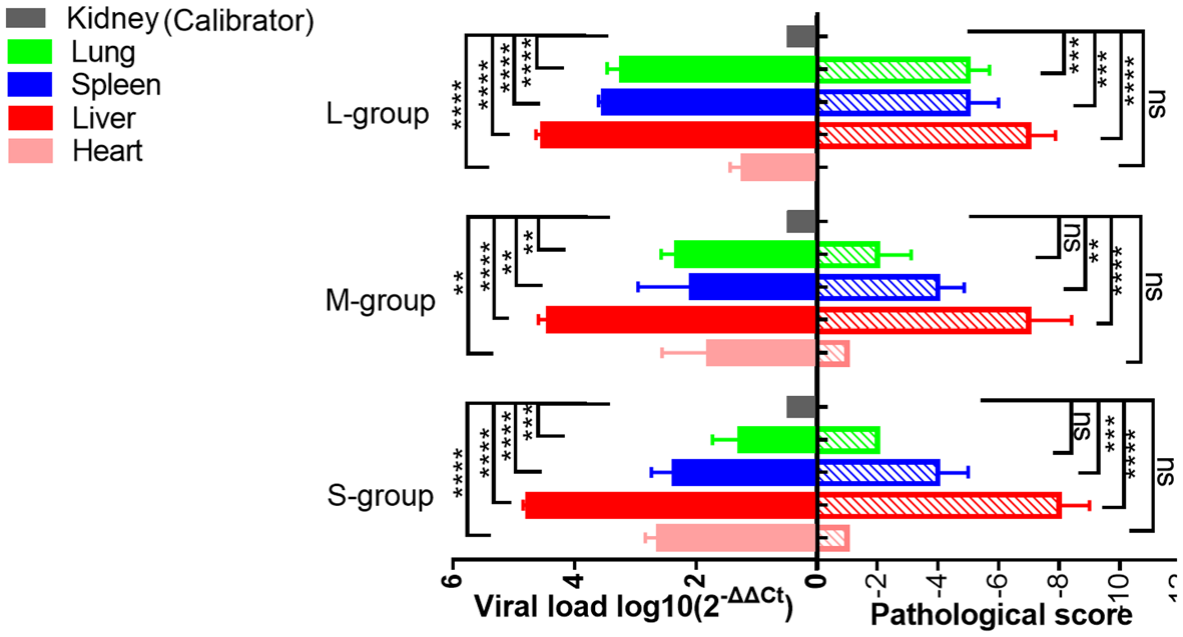
Column representation of visceral viral loads and a pathological scores of rabbits infected with GI.2. The viral load was calculated according to the formula 2^−ΔΔCT^. The higher the ΔCt value is, the lower the 2^−ΔΔCT^ value is. The kidney with the highest ΔCt value and the lowest expression of GI.1 was selected as Calibrator. Different asterisks indicate significant differences, *(*p* < 0.05), **(*p* < 0.01), ***(*p* < 0.001), ****(*p* < 0.0001); ns means the difference is not significant (*p* > 0.05).

As shown in Fig. 6, There was a positive correlation between the the viral loads and lesion scores of the viscera after GI.2 challenge. Compared with the hearts of the other groups, the hearts of adult rabbits had the lowest viral load and no lesions. The viral load in the liver of kittens was higher than that in subadult and adult rabbits, and the lesion score was the highest. The viral load of the spleen in adult rabbits was much higher than that in kittens and subadult rabbits, and the lesion score was also higher. The lung viral load in adult rabbits was higher than that in kittens and subadult rabbits, and the lesion score was also the highest. No apparent lesions were found in the kidneys of any of the rabbits in GI.2 challenge group.

## Discussion

At present, lagoviruses still cannot passage continuously in the cell culture system. In most of the previous challenge studies, which used hemagglutination (HA) units to quantify the amount of virus in inoculants, we assumed that HA was only an indirect indication of infection dose. Titration of inoculants is necessary in order to more accurately compare the pathogenicity of two lagoviruses under the same conditions.

Pathological changes and viral load after GI.2 infection have been widely reported elsewhere^16–19^. In contrast to several previous studies, GI.1 and GI.2 Chinese strains were used to infect rabbits of three different ages. In our experiments, we found that GI.2 had a mortality rate of 100% in rabbits of all ages, but kittens were more susceptible to GI.2 infection, with a short death time and a rapid course of the disease. The mortality rate of GI.1 was 100% (12/12) in rabbits over 90 days of age but 0 (0/6) in kittens.

The liver is the main organ invaded by GI.1/GI.2. Liver failure caused by degeneration, necrosis, or apoptosis of hepatocytes, is the main cause of acute death in rabbits. GI.1/GI.2 first appeared in hepatocytes, Kupfern cells, and sinus wall endothelial cells and then spread to other cells. During the course of disease development, hepatocytes and vascular endothelial cells had been found to be preferentially infected and damaged^25^. This finding is consistent with the results of our study.

We found that disease progression was influenced by both age and presence of the virus. The average death time of rabbits infected with GI.2 was shorter than that of rabbits infected with GI.1. Adult rabbits infected with GI.1 had the shortest and most acute course of the disease. The kittens were infected with GI.2 during the most urgent period of the disease. This trend was also reflected in the time to the onset of pyrexia and in the progression of viremia.

We observed that GI.1 was not lethal to the kittens. Virus copies in the blood and saliva of kittens infected with GI.1 first increased and then decreased. A study by Marques^26^ showed that kittens (4 weeks of age) are naturally resistant to GI.1, which is associated with a rapid and effective inflammatory response in the liver after GI.1 infection in kittens, with almost no hepatocytes infected by GI.1.

In our study, we found that the target organs of GI.1 and GI.2 were the liver, but the organs colonized by the two viruses differed. Notably, GI.1 did not colonize the heart, whereas GI.2 did. Meanwhile, GI.2 did not appear to colonise the kidney of infected rabbits in terms of viral load, unlike those infected by GI.1.

Comparing viral load in the spleen, we found that GI.1 in the spleen of kittens had the lowest viral load, while GI.2 in the spleen of kittens was higher than that of subadult rabbits, and loads of GI.1 and GI.2 in the spleen were higher in adult rabbits than in rabbits of other ages. The viral loads of GI.1 and GI.2 in the lungs were proportional to the age of rabbits. The older the rabbit was, the higher the viral load.

Previous studies focused on disease progression and pathological damage to the liver, spleen, and lungs following GI.1/GI.2 infection. In this study, we conducted animal trials under the same conditions (both viruses simultaneously, rabbit age groups, same virus dose and inoculation route), which enriched the research on the pathogenicity of GI.1 and GI.2. We found that liver and spleen were the main organs of viral colonization during the acute progression of RHD. At the same time, the age of rabbits also has an effect on the viral colonization of lungs and spleen. Future studies can continue to explore why the spleen and lungs of adults are more likely to become the organs of GI.1/GI.2 colonization than those of subadults and kittens. In addition, possibly due to the high dose of infection, we found that the heart and kidney were the most unaffected organs in the rapid course of disease in rabbits of any age infected with either GI.1 or GI.2, respectively. We believe that it will be a future research to explore why GI.1 and GI.2 have less damage to the kidney and heart, and whether the longer disease course has new effects on the internal organs of rabbits.

## Supplementary Information

Supplementary Information.

## Data Availability

The datasets generated during and/or analyzed during the current study are available from the corresponding author on reasonable request. All data generated or analyzed during this study are included in this published article.
