# Supplementary material for: The pathogenicity comparison of Lagovirus europaeus GI.1 and GI.2 strains in China by using relative quantitative assay
Source: Sci Rep. 2022 Nov 28;12:20518. doi: 10.1038/s41598-022-25118-0 (PMC9705280; doi:10.1038/s41598-022-25118-0)
Supplement: Supplementary file 1 — Supplementary Information. [file 41598_2022_25118_MOESM1_ESM.docx]

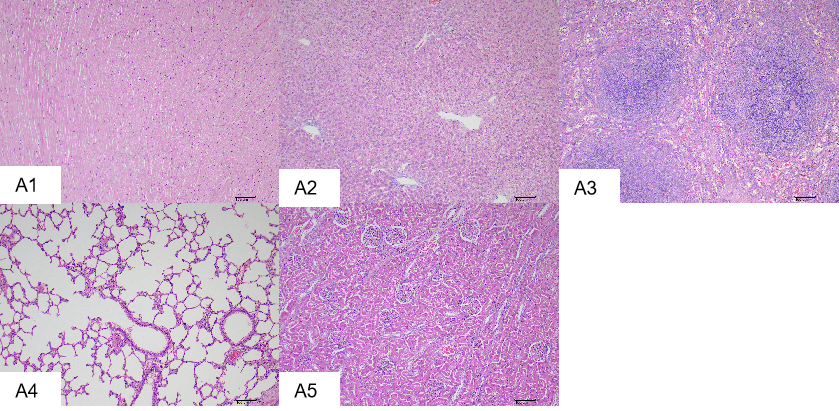


Figure S1. Observation of HE staining results of viscera in the control group

A1: Myocardial tissue; A2: Liver tissue; A3: Spleen tissue; A4: Lung tissue; A5: Kidney tissue

(H&E stain, 200 ×magnification)

The tissues of each organ were closely arranged, with no abnormal structure or morphology


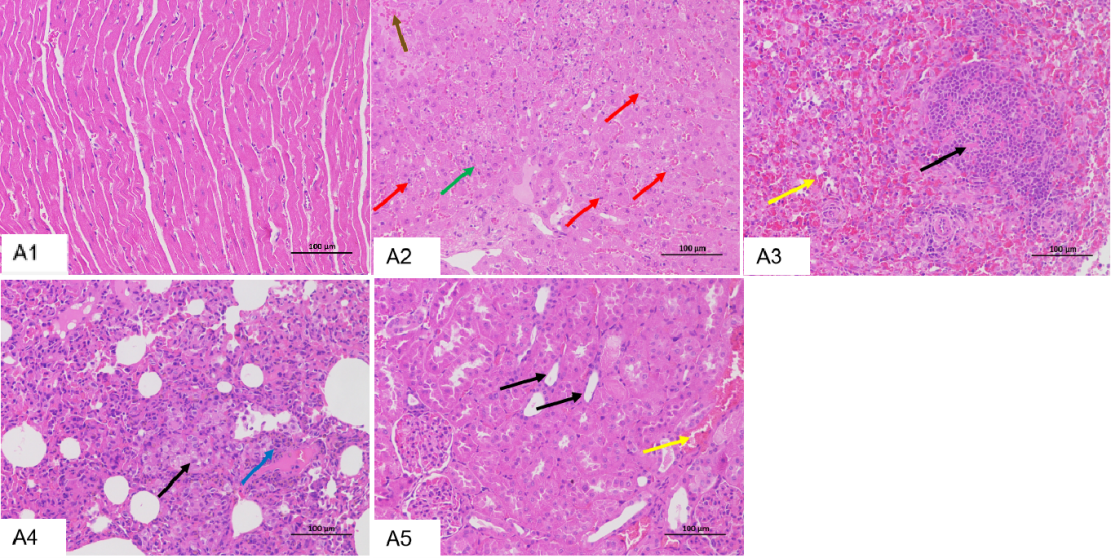


Figure S2. Histological observation of HE staining results of viscera in the GI.1-Adult group

A1: Myocardial tissue; A2: Liver tissue; A3: Spleen tissue; A4: Lung tissue; A5: Kidney tissue

(H&E stain, 200 × magnification)

A1: Myocardial tissue was uniformly stained, no significant inflammatory changes were observed . A2: Hepatic sinusoids were congested (green arrows), hepatocytes were arranged irregularly, punctate necrosis of hepatocytes was observed in many places, cytopyknosis was deeply stained, fragmented or dissolved (red arrows), erythrocytes were observed in many places of bleeding (brown arrows).

A3: The white pulp is largely reduced in number and size, and the number of lymphocytes is largely reduced. Eosinophils are seen in the white pulp (black arrows), and extensive congestion in the red pulp (yellow arrows).

A4: Severe thickening of the alveolar wall and widening of the alveolar septum were observed in large areas of the lung, accompanied by granulocyte infiltration (blue arrows) and occasional small focal infiltration of macrophages (black arrows).

A5: Vascular congestion (yellow arrows) and flattening of the tubular epithelium (black arrows) are seen in many places in the kidney.


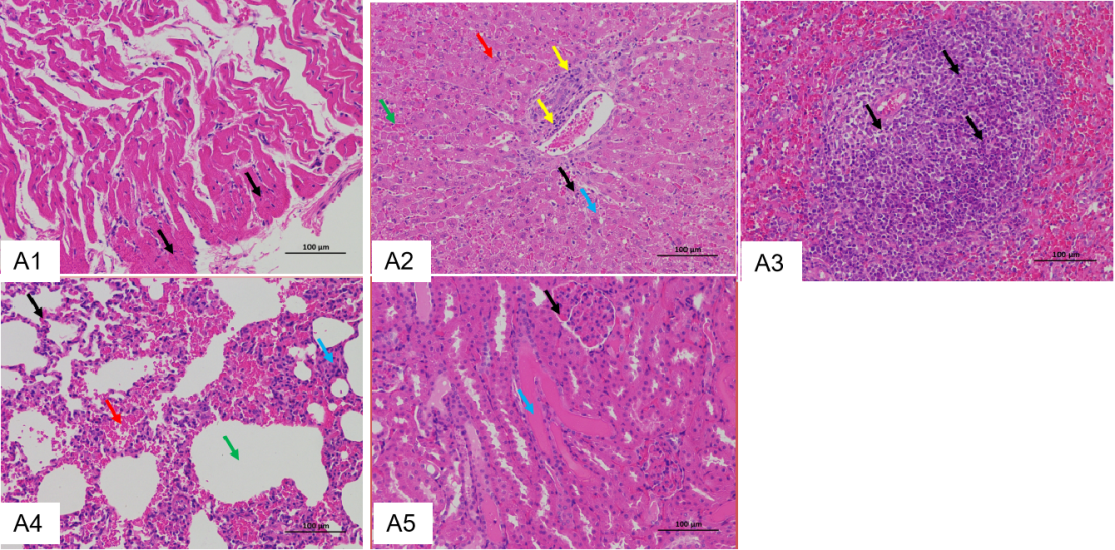


Figure S3. Histological observation of HE staining results of viscera in the GI.1-Subadult group

A1: Myocardial tissue; A2: Liver tissue; A3: Spleen tissue; A4: Lung tissue; A5: Kidney tissue

(H&E stain, 200 × magnification)

A1: A few cardiomyocytes are necrotic (black arrows)

A2: liver cells (black arrow), cytoplasm are seen in small fat cavitation (blue arrow), a disorder liver cells and liver sinus congestion (green arrow), more than the amount of liver cells dotted necrosis (red arrows), nucleus pycnosis, fracture, disappear, portal area around mild fibrosis, scattered lymphocytes infiltration (yellow arrow)

A3: Small amounts of necrotic cell debris are seen (black arrows)

A4: Slight thickening of the alveolar septum (blue arrows), slight bleeding of the local alveolar space (red arrows), and slight dilation of some alveolar Spaces (green arrows)

A5: Hyperemia and dilatation of glomerular capillaries in renal tissue (black arrows), eosinophilic protein fluid in the lumen of individual renal vesicles and tubules (blue arrows)

**
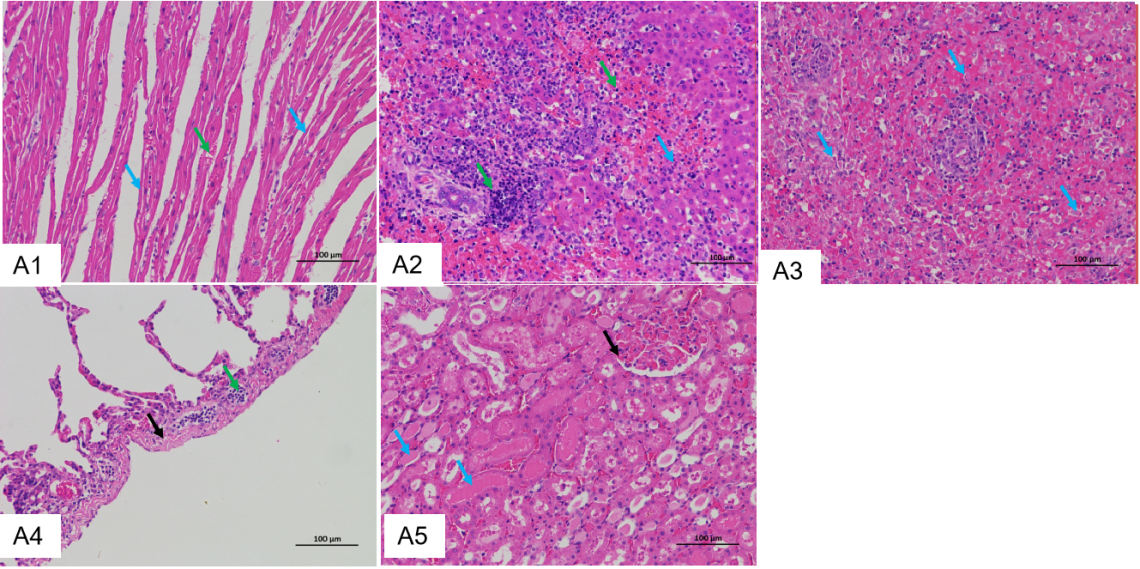
**

Figure S4. Histological observation of HE staining results of viscera in the GI.1-Kitten group

A1: Myocardial tissue; A2: Liver tissue; A3: Spleen tissue; A4: Lung tissue; A5: Kidney tissue

(H&E stain, 200 × magnification)

A1: Slight atrophy of cardiomyocytes (blue arrows) and hyperemia of interstitial capillaries (green arrows)

A2: Multifocal hemorrhagic necrosis of hepatocytes (blue arrows) with more inflammatory cell infiltration (green arrows)

A3: The red pulp was bleeding, histiocytic and necrotic cell debris (blue arrows).

A4: Mild fibrous hyperplasia of the lung capsule (black arrows), with locally dilated lymphatic vessels filled with lymphocytes (green arrows)

A5: Hyperemia and dilation of glomerular capillaries (black arrows), eosinophilic protein fluid in the lumen of more renal tubules (blue arrows)


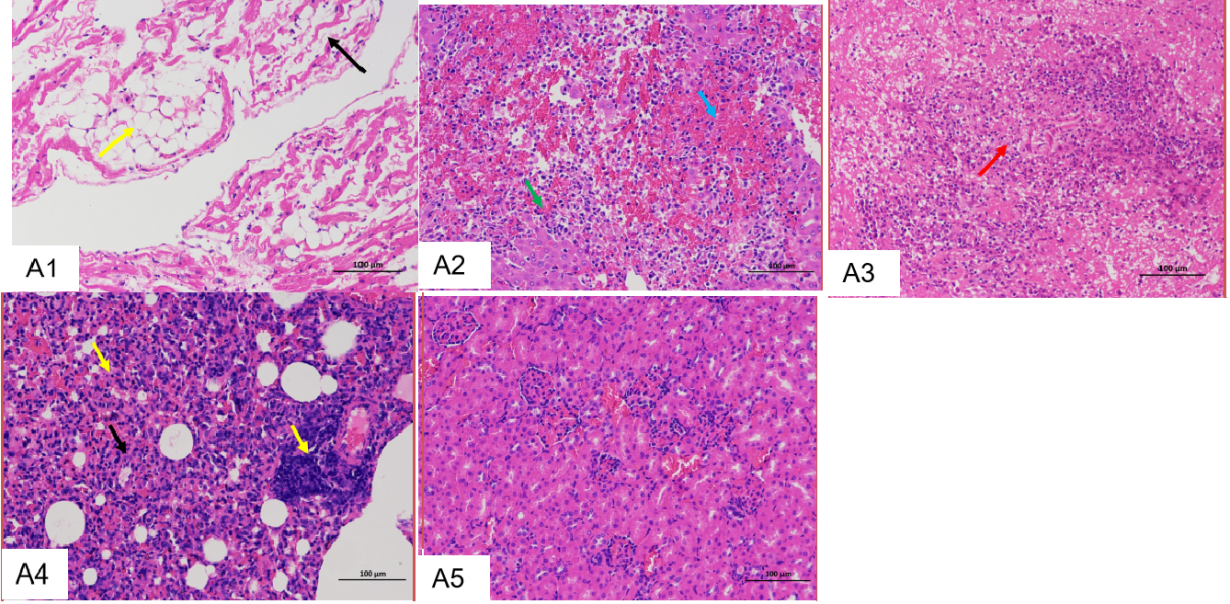


Figure S5. Histological observation of HE staining results of viscera in the GI.2-Adult group

A1: Myocardial tissue; A2: Liver tissue; A3: Spleen tissue; A4: Lung tissue; A5: Kidney tissue

(H&E stain, 200 × magnification)

A1: Cardiomyocytes were loosely arranged and spaced out (black arrows), with small areas of adipocyte infiltration (yellow arrows).

A2: Hepatic lobule structure disorder, hemorrhagic necrosis of hepatocytes (blue arrow), and more inflammatory cell infiltration (green arrow).

A3: There is diffuse necrosis of the splenic tissue, and a large number of cells with pyknotic nuclei are hyperchromatic, fragmented, or dissolved, and fused with the surrounding tissue as an unstructured eosinophilic material (red arrows).

A4: Alveolar space narrowing or even disappearance (black arrows) with small inflammatory cell infiltration (yellow arrows).

A5: There was no obvious abnormality in morphology.


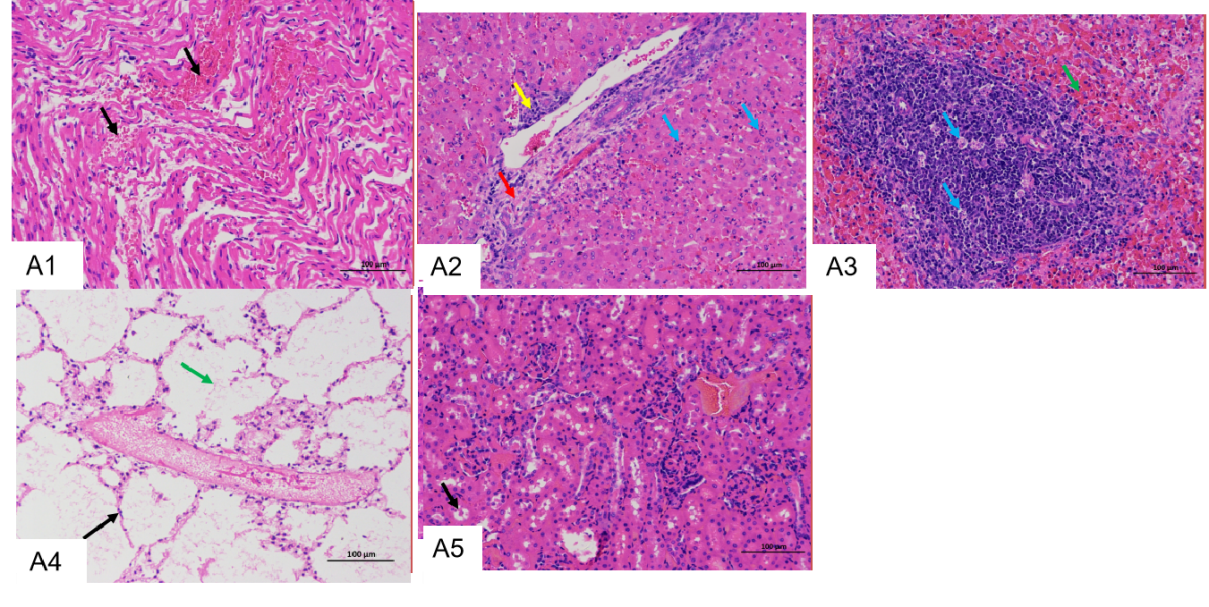


Figure S6. Histological observation of HE staining results of viscera in the GI.2-Subadult group

A1: Myocardial tissue; A2: Liver tissue; A3: Spleen tissue; A4: Lung tissue; A5: Kidney tissue

(H&E stain, 200 × magnification)

A1: Occasional local mild interstitial bleeding (black arrow).

A2: More punctate necrosis of hepatocytes (blue arrows), hyperplasia of connective tissue and bile ducts in the portal area (red arrows), with scattered lymphocytic infiltration (yellow arrows).

A3: Moderate damage to the white pulp, visible lymphocyte necrosis (blue arrow), moderate congestion and bleeding in the red pulp (green arrow).

A4: Numerous eosinophilic floc is seen in the alveolar lumen (green arrow) and numerous hyperchromatic nuclei in the epithelial cells of the alveolar wall (black arrow).

A5: There was no obvious abnormality in morphology and structure.


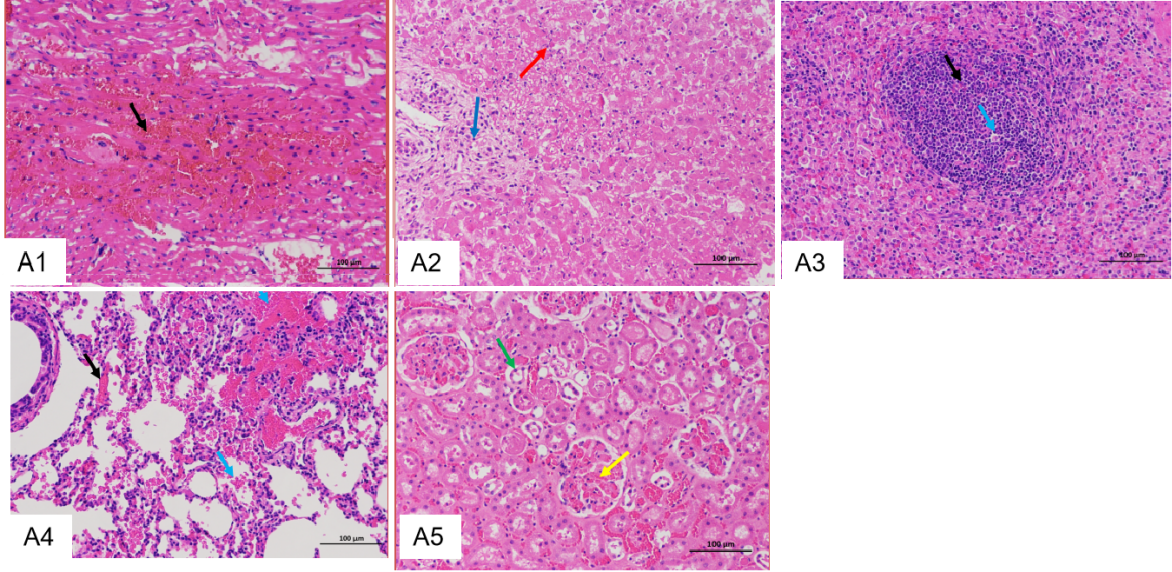


Figure S7. Histological observation of HE staining results of viscera in the GI.2-Kitten group

A1: Myocardial tissue; A2: Liver tissue; A3: Spleen tissue; A4: Lung tissue; A5: Kidney tissue

(H&E stain, 200 × magnification)

A1: A regular arrangement of cardiomyocytes with localized mild interstitial bleeding (black arrow).

A2: A significant amount of hepatocyte pyknosis was stained, fragmented, or dissolved (red arrows), and inflammatory cell infiltration was seen around the blood vessels (blue arrows).

A3: Moderate damage to the white pulp, reduced in number, reduced in size of remaining white pulp (black arrow), and lymphocytic necrosis (blue arrow).

A4: There is extensive capillary congestion in the alveolar wall (black arrows), and multifocal bleeding of varying degrees in the alveolar space (blue arrows).

A5: The glomerular capillaries are congested (yellow arrows), and a few tubular epithelial cells are shed (green arrows).

Table S1 Details of the RT-qPCR method

| Primers | Standard curve and  primer amplification efficiency | thermal cycles  conditions | | The reaction  volume (20μL) |
| --- | --- | --- | --- | --- |
| GI.2-F/R | Ct=–3.347×lg copies＋39.99  Amplification efficiency:99.0% | | 95℃ 30s；  95℃5 s，55.4℃25 s，72℃30 s(40 cycles) | **·**10μL of qPCR Mix, 1μL (5μmol/L) of each of the primers, 2μL of the template and 6μL ddH2O. |
|  |  | |  |  |
| GI.1- F/R | Ct=–3.43×lg copies＋53.10  Amplification efficiency:95.6% | | 95℃ 30s；  95℃5 s，57.1℃10 s，72℃30 s(40 cycles) | **·**10μL of qPCR Mix, 0.8μL (10μmol/L) of each of the primers, 2μL of the template and 6.4μL ddH2O. |
|  |  | |  |  |
| *β-actin*- F/R | Ct=–3.175×lg copies＋40.88  Amplification efficiency:98.1% | | 95℃ 30s；  95℃5 s，60℃10 s，  72℃30 s(40 cycles) | **·**10μL of qPCR Mix, 1μL (10μmol/L) of each of the primers, 2μL of the template and 6μL ddH2O. |
|  |  | |  |  |

Table S2. Pathological scores of hearts

| Groups | Pathological changes of heart | | | |
| --- | --- | --- | --- | --- |
|  | Bleeding | Congestion | Necrosis | Total |
| GI.2-Kitten | 1 | 0 | 0 | 1 |
| GI.2-Subadult | 1 | 0 | 0 | 1 |
| GI.2-Adult | 0 | 0 | 0 | 0 |
| GI.1-Kitten | 0 | 0 | 1 | 1 |
| GI.1-Subadult | 0 | 0 | 1 | 1 |
| GI.1-Adult | 0 | 0 | 0 | 0 |

Notes: 1. According to myocardial tissue arrangement, from "no lesions or very few lesions (0)" to "no normal tissue structure (4)"; 2. According to the interstitial bleeding, from "no or very little bleeding (0)" to "Extremely serious interstitial bleeding (4)"; 3. According to the degree of myocardial cell necrosis, from "no or very little necrosis (0)" to "a large number of necrotic lesions (4)".

Table S3. Pathological scores of livers

| Groups | Pathological changes of liver | | | | |
| --- | --- | --- | --- | --- | --- |
|  | Necrosis | Inflammatory cell infiltration | Congestion | Cell degeneration | Total |
| GI.2-Kitten | 4 | 2 | 2 | 0 | 8 |
| GI.2-Subadult | 3 | 2 | 2 | 0 | 7 |
| GI.2-Adult | 3 | 4 | 0 | 0 | 7 |
| GI.1-Kitten | 2 | 2 | 0 | 0 | 4 |
| GI.1-Subadult | 3 | 1 | 2 | 2 | 8 |
| GI.1-Adult | 3 | 3 | 3 | 0 | 9 |

Notes: 1. According to the interstitial bleeding, from "no or very little bleeding(0)" to "Extremely serious interstitial bleeding(4)"; 2. According to the necrosis of liver cell , from "no necrosis or very little necrosis (0)" to "a large number of liver cell necrosis lesions (4)"; 3. According to the infiltration of inflammatory cell , from "no inflammatory cells or very few inflammatory cell infiltration (0)" to "a large number of inflammatory cell infiltration (4)"; 4. According to the degree of hepatocyte degeneration: from "no cell degeneration or very few cells degeneration (0)" to "a large number of fat vacuoles in the cytoplasm (4)".

Table S4. Pathological scores of spleens

| Groups | Pathological changes of spleen | | | | |
| --- | --- | --- | --- | --- | --- |
|  | Necrosis | A decrease in the white pulp | Congestion | Inflammatory cell infiltration | Total |
| GI.2-Kitten | 4 | 0 | 0 | 0 | 4 |
| GI.2-Subadult | 2 | 2 | 0 | 0 | 4 |
| GI.2-Adult | 4 | 0 | 0 | 1 | 5 |
| GI.1-Kitten | 1 | 1 | 0 | 0 | 2 |
| GI.1-Subadult | 0 | 2 | 0 | 2 | 4 |
| GI.1-Adult | 0 | 1 | 4 | 1 | 6 |

Notes: 1. According to the interstitial bleeding, from "With no or very little bleeding(0)" to "Extremely serious interstitial bleeding(4)"; 2. According to the degree of spleen cell necrosis, from "no or very little necrosis (0)" to "a large number of spleen cell necrosis lesions (4)"; 3. According to the degree of inflammatory cell infiltration, from "no inflammatory cells or very few inflammatory cell infiltration (0)" to "a large number of inflammatory cell infiltration (4)"; 4. According to the degree of vascular congestionfrom "there is no or little congestion(0)" to "there is extremely serious or massive congestion(4)".

Table S5. Pathological scores of lungs

| Groups | Pathological changes of lung | | | | | |
| --- | --- | --- | --- | --- | --- | --- |
|  | Congestion | Thickening of the alveolar wall | Inflammatory cell infiltration | Bleeding | Alveolar expansion | Total |
| GI.2-Kitten | 2 | 0 | 0 | 0 | 0 | 2 |
| GI.2-Subadult | 2 | 0 | 0 | 0 | 0 | 2 |
| GI.2-Adult | 1 | 2 | 1 | 1 | 0 | 5 |
| GI.1-Kitten | 0 | 0 | 1 | 0 | 0 | 1 |
| GI.1-Subadult | 1 | 1 | 0 | 1 | 0 | 3 |
| GI.1-Adult | 0 | 4 | 2 | 0 | 0 | 6 |

Notes: 1. According to the degree of capillary congestion in the alveolar wall, from "no or little congestion (0)" to"extremely serious or massive congestion(4)"; 2. According to the degree of inflammatory cell infiltration, from "no inflammatory cells or very few inflammatory cell infiltration (0)" to "a large number of inflammatory cell infiltration (4)"; 3. According to the interstitial bleeding, from "no or very little bleeding (0)" to "Extremely serious interstitial bleeding(4)"; 4. According to the degree of alveolar wall thickening, from "no or mild thickening of alveolar wall (0)" to "severe thickening of alveolar wall, narrowing or even disappearance of alveolar cavity (4)".

Table S6. Pathological scores of kidneys

| Groups | Pathological changes of kidney | | |
| --- | --- | --- | --- |
|  | Congestion | Flattening of renal tubular epithelium | Total |
| GI.2-Kitten | 0 | 0 | 0 |
| GI.2-Subadult | 0 | 0 | 0 |
| GI.2-Adult | 0 | 0 | 0 |
| GI.1-Kitten | 2 | 0 | 2 |
| GI.1-Subadult | 2 | 0 | 2 |
| GI.1-Adult | 2 | 1 | 3 |

Notes: 1, according to the degree of capillary blood vessel congestion, from "there is no or little congestion (0)" to "there is extremely serious or massive congestion(4)"; 2. According to the flattening degree of renal tubular epithelium, from "no renal tubular epithelial flattening (0)" to "a large number of renal tubular epithelial flattening (4)"

Table S7. Distribution of viral load in the viscera of GI.1 artificial infection rabbits

| [Viscera](C:/Users/%E6%B6%82%E8%97%A4/AppData/Local/youdao/dict/Application/9.1.0.0/resultui/html/index.html#/javascript:;)  Groups | | Heart（Calibrator） | Liver | Spleen | Lung | Kidney |
| --- | --- | --- | --- | --- | --- | --- |
| Kittens | R1-S1 | 0 | 1.81 | 0.54 | 0.83 | 2.02 |
|  | R1-S2 | 0 | 1.96 | 0.95 | 0.35 | 2.19 |
|  | R1-S3 | 0 | 2.05 | 1.1 | 0.36 | 1.98 |
|  | R1-S4 | 0 | 1.79 | 0.76 | 0.77 | 2.05 |
|  | R1-S5 | 0 | 1.99 | 0.97 | 0.45 | 1.92 |
|  | R1-S6 | 0 | 2.04 | 0.91 | 0.31 | 2.21 |
| Subadult rabbits | R1-M1 | 0 | 3.49 | 2.38 | 1.92 | 1.73 |
|  | R1-M2 | 0 | 3.62 | 2.68 | 1.9 | 1.39 |
|  | R1-M3 | 0 | 3.69 | 2.57 | 1.97 | 1.58 |
|  | R1-M4 | 0 | 3.25 | 2.71 | 1.87 | 1.7 |
|  | R1-M5 | 0 | 3.68 | 2.33 | 1.88 | 1.36 |
|  | R1-M6 | 0 | 3.87 | 2.57 | 2.04 | 1.55 |
| Adult rabbits | R1-L1 | 0 | 4.75 | 4.13 | 2.49 | 1.41 |
|  | R1-L2 | 0 | 4.67 | 4.24 | 2.78 | 1.87 |
|  | R1-L3 | 0 | 4.74 | 4.14 | 2.42 | 2.16 |
|  | R1-L4 | 0 | 4.52 | 4.21 | 2.72 | 2.05 |
|  | R1-L5 | 0 | 4.66 | 4.17 | 2.44 | 1.81 |
|  | R1-L6 | 0 | 4.98 | 4.13 | 2.53 | 1.58 |

GI.1 RT-qPCR and β-actin RT-qPCR were used to measure the Ct values of target genes, and the results were calculated according to 2^－ΔΔCt^. Heart was selected as Calibrator to realize data normalization of other organs, and the results of visceral viral load were converted to log10（2^－ΔΔCt^).

Table S8. Distribution of viral load in the viscera of GI.2 artificial infection rabbits

| [Viscera](C:/Users/%E6%B6%82%E8%97%A4/AppData/Local/youdao/dict/Application/9.1.0.0/resultui/html/index.html#/javascript:;)  Groups | | Heart | Liver | Spleen | Lung | Kidney（Calibrator） |
| --- | --- | --- | --- | --- | --- | --- |
| Kittens | R2-S1 | 2.84 | 4.8 | 2.79 | 1.62 | 0 |
|  | R2-S2 | 2.41 | 4.83 | 2.2 | 0.76 | 0 |
|  | R2-S3 | 2.61 | 4.68 | 2.1 | 1.45 | 0 |
|  | R2-S4 | 2.75 | 4.81 | 2.51 | 1.32 | 0 |
|  | R2-S5 | 2.55 | 4.79 | 2.1 | 1.45 | 0 |
|  | R2-S6 | 2.56 | 4.71 | 2.47 | 1.05 | 0 |
| Subadult rabbits | R2-M1 | 0.93 | 4.49 | 2.55 | 2.47 | 0 |
|  | R2-M2 | 2.02 | 4.25 | 2.62 | 2.02 | 0 |
|  | R2-M3 | 2.42 | 4.56 | 1.07 | 2.47 | 0 |
|  | R2-M4 | 1.55 | 4.51 | 2.41 | 2.12 | 0 |
|  | R2-M5 | 1.04 | 4.55 | 2.01 | 2.36 | 0 |
|  | R2-M6 | 2.78 | 4.24 | 1.82 | 2.48 | 0 |
| Adult rabbits | R2-L1 | 1.01 | 4.63 | 3.43 | 2.95 | 0 |
|  | R2-L2 | 1.44 | 4.4 | 3.61 | 3.33 | 0 |
|  | R2-L3 | 1.23 | 4.54 | 3.51 | 3.4 | 0 |
|  | R2-L4 | 1.21 | 4.6 | 3.48 | 3.3 | 0 |
|  | R2-L5 | 1.35 | 4.46 | 3.41 | 2.7 | 0 |
|  | R2-L6 | 1.12 | 4.51 | 3.67 | 3 | 0 |

GI.2 RT-qPCR and β-actin RT-qPCR were used to measure the Ct values of target genes, and the results were calculated according to 2^－ΔΔCt^. Kidney was selected as Calibrator to realize data normalization of other organs, and the results of visceral viral load were converted to log10（2^－ΔΔCt^).
